# Identification, Synthesis, Conformation and Activity of an Insulin-like Peptide from a Sea Anemone

**DOI:** 10.3390/biom11121785

**Published:** 2021-11-29

**Authors:** Michela L. Mitchell, Mohammed Akhter Hossain, Feng Lin, Ernesto L. Pinheiro-Junior, Steve Peigneur, Dorothy C. C. Wai, Carlie Delaine, Andrew J. Blyth, Briony E. Forbes, Jan Tytgat, John D. Wade, Raymond S. Norton

**Affiliations:** 1 Medicinal Chemistry, Monash Institute of Pharmaceutical Sciences, Monash University, 381 Royal Parade, Parkville, VIC 3052, Australia; dorothy.wai@monash.edu; 2 Sciences Department, Museum Victoria, G.P.O. Box 666, Melbourne, VIC 3001, Australia; 3 Biodiversity and Geosciences, Queensland Museum, P.O. Box 3000, South Brisbane, QLD 4101, Australia; 4 Florey Institute of Neuroscience and Mental Health, University of Melbourne, Parkville, VIC 3010, Australia; akhter.hossain@unimelb.edu.au (M.A.H.); feng.lin@florey.edu.au (F.L.); john.wade@florey.edu.au (J.D.W.); 5 School of Chemistry, University of Melbourne, Parkville, VIC 3010, Australia; 6 Toxicology and Pharmacology, University of Leuven, O&N 2, Herestraat 49, P.O. Box 922, 3000 Leuven, Belgium; ernesto.lopes@kuleuven.be (E.L.P.-J.); steve.peigneur@kuleuven.be (S.P.); jan.tytgat@kuleuven.be (J.T.); 7 Flinders Health and Medical Research Institute, Flinders University, Bedford Park, SA 5042, Australia; carlie.delaine@flinders.edu.au (C.D.); andrew.blyth@flinders.edu.au (A.J.B.); briony.forbes@flinders.edu.au (B.E.F.); 8 ARC Centre for Fragment-Based Design, Monash University, Parkville, VIC 3052, Australia

**Keywords:** *Oulactis*, cnidaria, peptide synthesis, ion channel, invertebrates, insulin

## Abstract

The role of insulin and insulin-like peptides (ILPs) in vertebrate animals is well studied. Numerous ILPs are also found in invertebrates, although there is uncertainty as to the function and role of many of these peptides. We have identified transcripts with similarity to the insulin family in the tentacle transcriptomes of the sea anemone *Oulactis* sp. (Actiniaria: Actiniidae). The translated transcripts showed that these insulin-like peptides have highly conserved A- and B-chains among individuals of this species, as well as other Anthozoa. An *Oulactis* sp. ILP sequence (IlO1_i1) was synthesized using Fmoc solid-phase peptide synthesis of the individual chains, followed by regioselective disulfide bond formation of the intra-A and two interchain disulfide bonds. Bioactivity studies of IlO1_i1 were conducted on human insulin and insulin-like growth factor receptors, and on voltage-gated potassium, sodium, and calcium channels. IlO1_i1 did not bind to the insulin or insulin-like growth factor receptors, but showed weak activity against K_V_1.2, 1.3, 3.1, and 11.1 (hERG) channels, as well as Na_V_1.4 channels. Further functional studies are required to determine the role of this peptide in the sea anemone.

## 1. Introduction

The role of insulin as a key anabolic hormone that promotes the absorption of glucose from the blood into liver, fat, and skeletal muscle cells in vertebrates is well documented [1,2,3]. In contrast, the presence and role of insulin-like peptides (ILPs) in invertebrates, especially those found in the marine environment, are less well understood. The characterized invertebrate ILPs perform an array of functions, which vary depending upon their tissue of origin. Insect ILPs (e.g., from *Drosophila melanogaster*) are implicated in multiple functions, including regulating metabolism, growth, reproduction, and longevity [4,5,6,7]. For example, bombyxin A-1, a silk moth (*Bombyx mori*) ILP, is produced in the brain to activate the prothoracic glands to produce ecdysone (a moulting hormone) for growth development [8]. The nematode worm *Caenorhabditis elegans* ILPs act as agonists and antagonists towards the insulin-like growth factor signaling pathway [9]. Marine ILPs have been documented in molluscs; in the Californian sea hare *Aplysia californica* ILPs have a role in metabolism [10]. In the Pacific oyster (*Crassostrea gigas*), ILPs are involved in sexual maturation [11]. ILPs have also been identified in the purple sea urchin (*Strongylocentrotus purpratus*) and the Eastern rock lobster (*Sagmariasus verreauxi*) [12,13].

More recently, marine invertebrate ILPs have been identified in the venom of two cone snails, *Conus geographus* and *C. tulipus* [14]. The snails release the ILPs into the water column amongst schools of fish. The fish succumb to a hypoglycemic stupor referred to as a “nirvana cabal”, whereupon the snail extends its mouth like a fishing net to capture the fish prior to venom injection [14,15,16]. This intriguing discovery of an ILP in cone snail venom with a role in prey capture has stimulated the search for additional functions and biological applications of ILPs in invertebrates.

One motivation for investigating marine ILPs is their potential anti-diabetic properties which may be translatable into pharmaceuticals [16]. Several marine organisms have been documented to contain anti-diabetic compounds, including sea anemones [17]. Pascual et al. [18] demonstrated that aqueous crude extracts from the sea anemones *Bunodosoma granuliferum* and *Bartholomea annulata* inhibit porcine dipeptidyl peptidase IV (DPPIV). Inhibition of this enzyme results in a reduction of glucagon and blood glucose levels and may therefore be useful as an alternative treatment for diabetes. The near-explosive growth in genomic information has led to the identification of large numbers of insulin-like peptides (ILPs) in many species of mammals, insects and viruses [19,20,21]. As an example, *Caenorhabditis elegans* is predicted to have, remarkably, no less than 40 different insulins [22]. Many of these peptides share the characteristic two-chain (A and B) and canonical three-disulfide-bond structure of human insulin. However, some possess single-chain structures containing three disulfide bonds, and some do not include an A-chain intramolecular disulfide. Other ILPs contain an additional interchain disulfide bond [23]. Depending on their general structure and number of disulfide bonds, they have been classified as α-type, β-type or γ-type [22,23]. 

The rapid growth in ‘omics’ studies includes the discovery of numerous ILPs in the genomes and transcriptomes of sea anemones, including for the model sea anemone *Nematostella vectensis* [24,25]. However, sea anemone ILPs have not been characterized functionally. Unlike other animals, cnidarians lack a circulatory and central nervous system. Therefore, the processing and physiological role(s) of ILPs in cnidarians are unclear and their functions are still to be determined, along with their potential as a source of therapeutics to treat diabetes.

Several insulin-like sequences were identified in the tentacle transcriptomes of the Australian speckled anemone (*Oulactis* sp.) [26], which may represent a new class of ILP. In this study we describe the synthesis of a representative ILP from this sea anemone and the results of activity assays against human insulin and insulin-like growth factor receptors, as well as a range of ion channels. 

## 2. Materials and Methods

### 2.1. Insulin-like Peptide Data and Bioinformatic Analysis

Translated amino acid sequences that share domain similarity within the insulin family (protein family PF00049) were identified during initial bioinformatic analysis of tentacle transcriptomes from the sea anemone *Oulactis* sp. (not yet formally described) [26]. These ILP sequences were deposited in the European Bioinformatics Institute—European Nucleotide Archive (EMBL-ENA) database under Project No. PRJEB34263 (Acc. No. LR700308-11). Following the initial identification, the search for ILPs was extended to two additional tentacle transcriptomes to determine whether the identified ILPs were present in other individuals of the species.

The search for ILPs was also extended to additional discrete tissue regions of *Oulactis* sp., including internal tissue (mesenterial filaments and gametic material) and external tissue (the frill and acrorhagi, located adjacent to the tentacles). Translated amino acid sequences were selected to match the search criteria of similarity to the insulin protein family PF00049 domain with an *E*-value: >1 × 10^−10^; full-length sequence i.e., including start and stop codons; and the presence of a signal peptide. The full-length translated amino acid sequence of insulin-like_Oulsp_1_i1 (simplified to IlO1_i1 for this study) (ENA-EMBL Acc. No. LR700308) formed the search basis in the NCBI non-redundant database (https://blast.ncbi.nlm.nih.gov/, accessed on May 2020) for other sequences with similarity. The NCBI-BLAST search was conducted online using the blastp algorithm (protein–protein BLAST). 

Selected sequences of characterized ILPs were downloaded from Uniprot (https://www.uniprot.org/, accessed on May 2020) for sequence-function comparison. Sequences were aligned using Clustal OMEGA online [27,28,29], and pairwise sequence alignments were conducted via the EMBL-ENA EMBOSS needle tool using the Needleman–Wunsch algorithm [30]. SignalP (4.1 server) [31] was used to predict the signal peptide and cleavage position for mature protein sequences. B-, C-, and A-chain cleavage sites were determined as per documented cleavage positions for human insulin (Lys-Arg, Arg-Arg) [32].

### 2.2. ILP Synthesis

**Figure 1:**
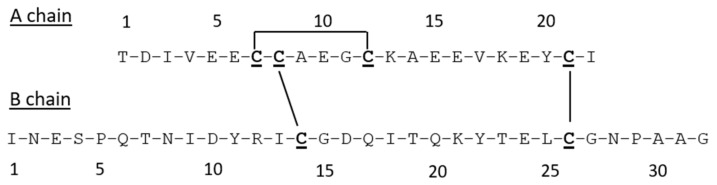
Truncated IlO1_i1 amino acid sequence synthesized using Fmoc solid-phase peptide synthesis chemistry. Cysteines are bolded and underlined, predicted disulfide bond connectivities are shown.

The sequence IlO1_i1 was selected for synthesis and subsequent functional characterization. For the purposes of the assembly, the N-terminal B-chain hexapeptide containing a pair of Cys residues was deleted, and the predicted canonical insulin Cys bonds were adopted (Figure 1). The deletion was undertaken for consistency with previously characterized insulin and ILP sequences, along with the regions of amino acid sequences known to be active on mammalian insulin receptors [16,33].

Each of the selectively S-protected A- and B-chains was assembled by Fmoc solid-phase peptide synthesis. For the A-chain peptide, S-protection was afforded by Trt (Cys7,12), Acm (Cys21), and *t*-Bu (Cys8). For the B-chain, Trt (Cys14) and Acm (Cys26) were used.

A-chain intramolecular disulfide oxidation: [Cys7,12 (S-thiol), Cys8(*t*-Bu), Cys21(Acm)] A-chain (66 mg, 25.5 µmol; Figure S4) was dissolved in 0.1 M Gly.NaOH (40.5 mL), and to this was added 1 mM 2-dipyridyl disulfide (DPDS) in MeOH (25.5 mL, 25.5 mmol). Oxidation was complete after 1 h, as monitored by analytical RP-HPLC. The peptide was isolated by preparative RP-HPLC and subsequent freeze drying to give 45.3 mg (44.0%) of purified [Cys21(Acm), Cys8(But)] A-chain (Figure S5).

[Cys8(Pyr), Cys21(Acm)] A-chain: Intramolecular disulfide-bonded [Cys8(But), Cys21(Acm)] A-chain (45.3 mg, 17.5 mmol) was converted to the Cys8 S-pyridinylsulfenyl (Pyr) form by treatment with DPDS in neat TFA (5.0 mL) containing anisole (0.5 mL) chilled to 0 °C, and then 5.0 mL TFMSA/TFA (1:5 *v*/*v*) was added and stirred for 20–30 min, maintaining the temperature at or below 0 °C. The peptide was then precipitated in ether and the pellet was suspended in 6 M GdnHCl for purification. The target peptide was isolated by preparative RP-HPLC to give 28.3 mg (61.1%) (Figure S6).

Combination of [Cys8(Pyr), Cys14(Acm)] A-chain with [Cys14(S-thiol), Cys26(Acm)] B-chain: A-chain peptide (25.9 mg, 9.6 mmol) was dissolved in 8 M GdnHCl (6 mL) and added to purified B-chain (34.5 mg, 9.6 mmol; Figure S7) in the same buffer (6 mL). The mixture was stirred vigorously at room temperature or 37 °C for each buffer, respectively, and the reaction was monitored by analytical RP-HPLC. After 30 min (or 24 h if using the GdnHCl buffer), the reaction was terminated by the addition of glacial acetic acid, and the target product was isolated by preparative RP-HPLC to give 22.7 mg (38.7%) (Figure S8).

The [Cys21(Acm)] A-chain/[Cys26(Acm)] B-chain (22.7 mg, 3.7 mmol) was dissolved in glacial acetic acid (16.7 mL) and 80 mM HCl (2.3 mL), and to this was added dropwise 3.7 mL of 20 mM iodine/acetic acid (74 mmol). After 1 h [34], the reaction was stopped by the addition of 3.7 mL of 20 mM ascorbic acid. Preparative RP-HPLC, as described above, was then used to isolate and purify the product, giving 15.6 mg (70.4%) in an overall total yield of 27.2%; the expected mass of the final product was calculated at [MH+] 5972.62, and that observed was [M/4+H] = 1493.91, [M/5+H] = 1195.33 (Figure S9).

### 2.3. Spectroscopic Studies

#### 2.3.1. Circular Dichroism Spectroscopy

Circular dichroism (CD) spectra were recorded on a Jasco J-1500 CD spectrometer from 300 to 180 nm with a 0.2 nm step size using a 1.0 s response time and 1.0 nm bandwidth in a quartz cuvette with a 0.2 cm path length. Insulin and IlO1_i1 peptide were resuspended in 10 mM sodium phosphate (pH 7.8) to a concentration of 0.2 mg/mL. To correct for background, the spectrum of buffer alone was subtracted from each sample spectrum. The machine units collected—θ in millidegrees, were converted to mean residue ellipticity (MRE), [θ] (degrees·cm^2^dmol^−1^residue^−1^). Helical content was calculated using the CDSSTR algorithm [35] for deconvolution against the reference protein database set SMP180. This program is available on the DICROWEB website (http://dichroweb.cryst.bbk.ac.uk/html/home.shtml, accessed on May 2020).

#### 2.3.2. Nuclear Magnetic Resonance (NMR) Spectroscopy

The NMR spectra of IlO1_i1 and Con-Ins G1 were acquired at 298 K on a Bruker Avance III spectrometer equipped with a TCI cryoprobe (Billerica, MA, USA). Quantities of 0.8 mg of IlO1_i1 and 1 mg of Con-Ins G1 were each dissolved in 500 µL H_2_O, and the pH was measured without adjustment before the addition of D_2_O (to a final concentration of 10%). ^1^H chemical shifts were referenced using dioxane (3.75 ppm), added after acquisition of the initial spectrum. Spectra were processed using Topspin (version 3.6.2).

### 2.4. Functional Studies

#### 2.4.1. Binding Assays

The ability to bind IlO1_i1 to the human insulin receptor (IR) and insulin-like growth factor receptor (IGF-1R) was measured in competition binding assays. BALB/c3T3 fibroblast cells overexpressing IGF-IR (P6 cells) [36] and IR-B cells (IGF-IR null mouse fibroblasts overexpressing the human insulin receptor isoform B) [37] were cultured in DMEM, 10% fetal calf serum, 1% penicillin/streptomycin, and G418 (250 µg/mL). IGF-IR and IR-B were solubilized from cells using lysis buffer (20 mM HEPES, 150 mM NaCl, 1.5 mM MgCl_2_, 10% (*v*/*v*) glycerol, 1% (*v*/*v*) Triton X-100, 1 mM EGTA (pH 7.5)) for 1 h at 4 °C, and lysates were centrifuged for 10 min at 3500 rpm. Solubilized IGF-1R or IR-B (100 µL) was used to coat each well of a white Greiner Lumitrac 600 plate previously coated with 24–31 anti-human IGF-1R antibody or 83-7 anti-human IR antibody [38,39]. Europium-labelled IGF-I or insulin (~3,000,000 counts) was added to wells with increasing concentrations of competitive ligand (IGF-I, insulin, or IlO1_i1 peptide) and incubated for 16 h at 4 °C. Wells were washed three times with 20 mM Tris, 150 mM NaCl, 0.1% (*v*/*v*) Tween 20, and then DELFIA enhancement solution (100 µL) was added. Time-resolved fluorescence was measured with 340 nm excitation and 612 nm emission filters using a Victor X4, 2030 Multilabel Reader (Perkin Elmer). All assays were repeated at least 3 times with 3 technical replicates each, except for the IlO1_i1 peptide binding IGF-IR, where 2 assays were conducted. Mean IC_50_ values were calculated using the statistical software package Prism v9.0.0 (GraphPad Software) after curve fitting with a nonlinear regression (one-site) model. 

#### 2.4.2. Ion Channel Assays

For the expression of K_V_ channels (mammalian rK_V_1.2, hK_V_1.3, rK_V_2.1, hK_V_3.1, rK_V_4.2, hK_V_7.2/hK_V_7.3, hK_V_10.1, hERG and Shaker IR from the fruit fly *Drosophila melanogaster*), Na_V_ channels (mammalian rNa_V_1.2, rNa_V_1.3, rNa_V_1.4, hNa_V_1.5, rNa_V_1.6, hNa_V_1.7, BgNa_V_ from the cockroach *Blattella germanica,* as well as the auxiliary subunits rβ1, hβ1), and the hCa_V_3.1 channel in *Xenopus* oocytes, the linearized plasmids were transcribed using the T7 or SP6 mMESSAGE-mMACHINE transcription kit (Ambion, Carlsbad, CA, USA). The harvesting of stage V–VI oocytes from an anaesthetized female *Xenopus laevis* frog was described previously [40] and was in compliance with the regulations of the European Union (EU) concerning the welfare of laboratory animals as declared in Directive 2010/63/EU. The use of *X. laevis* oocytes was approved by the Animal Ethics Committee of the KU Leuven with the licence number P186/2019. Oocytes were injected using a microinjector (Drummond Scientific, Broomall, PA, USA) with 4–50 nL of cRNA, depending on the channel subtype, and subsequently incubated in ND96 solution (96 mM NaCl, 2 mM KCl, 1.8 mM CaCl_2_, 2 mM MgCl_2_, and 5 mM HEPES, pH 7.4), supplemented with 50 mg/L gentamycin sulfate.

Two-electrode voltage-clamp recordings were performed at room temperature (18–22 °C) using a Geneclamp 500 amplifier (Molecular Devices, Downingtown, PA, USA) controlled by a pClamp data acquisition system (Axon Instruments, Union City, CA, USA). Whole cell currents from oocytes were recorded 1–7 days after injection. The bath and perfusion solutions were either the previously described ND96 (Na_V_ and K_V_ channels) or calcium-free ND96 supplemented with 10 mM BaCl_2_ (Ca_V_ channels). Toxins were applied directly to the bath. The resistances of both electrodes were kept between 0.8 and 1.5 MΩ. Currents were sampled at 20 kHz (Na_V_ channels) and 2 kHz (K_V_ and Ca_V_ channels) and filtered using a four-pole low-pass Bessel filter, at 1 kHz for sodium and 500 MHz for potassium and calcium channels, except for hERG, in which the currents were filtered at 1 kHz. Leak subtraction was performed using a P/4 protocol. K_V_1.x currents were evoked by 500 ms depolarizations to 0 mV followed by a 500 ms pulse to −50 mV, from a holding potential of −90 mV. K_V_2.1, K_V_3.1, and K_V_4.2 currents were elicited by 500 ms pulses to +20 mV from a holding potential of −90 mV. Current traces of the hERG channel were elicited by applying a +40 mV pre-pulse for 2 s, followed by a step of −120 mV for 2 s. Current traces of K_V_10.1 were elicited by 2 s depolarization to 0 mV, from a holding potential of −90 mV. Sodium current traces were evoked by a 100 ms depolarization to 0 mV. For Ca_V_ channels, current traces were elicited by 700 ms depolarizations to −20 mV from a holding potential of −90 mV.

## 3. Results

### 3.1. ILP Sequence Similarity

Several transcripts identified in the tentacle transcriptomes of three individuals of the speckled sea anemone (*Oulactis* sp.) [26] bore sequence similarity to the insulin family (Pfam PF00049) [41]. Analysis of individual transcripts showed that the A-chain is wholly conserved across individuals of the species (Supplementary Material, Figure S1). The B-chain is also highly conserved, varying at only one or two residues, including the penultimate residue (Supplementary Material, Figure S1).

**Figure 2:**
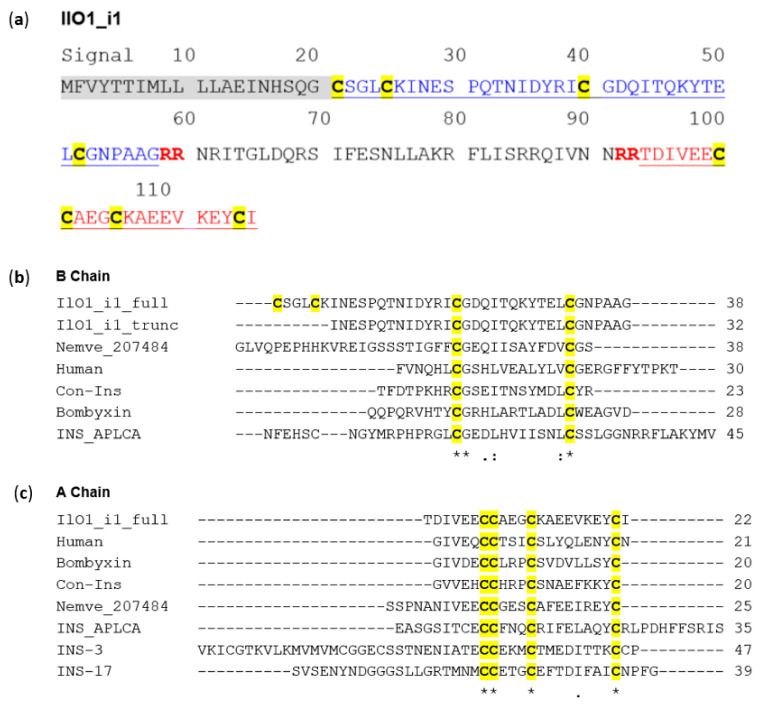
The full-length sequence of the sea anemone peptide ILP IlO1_i1 and alignment to characterized human and invertebrate insulin-like peptides (ILPs). (**a**) The full-length amino acid sequence of the sea anemone ILP. Signal peptide highlighted in grey, B-chain in blue, C-chain in black and A-chain in red. (**b**) Alignment of human and invertebrate B-chains to the predicted full-length B-chain of IlO1_i1 and the truncated sequence used for synthesis. (**c**) A-chain alignment of IlO1_i1, human and invertebrate ILPs. Sequences used in alignments with UniProt number supplied: Human insulin: P01308 [33]; Con-Ins: *Conus geographus*: A0A0B5AC95 [14]; Bombyxin: *Bombyx mori:* Q17192 [8]; Nemve_207484: *Nematostella vectensis*: A7S6C3 [44]; INS_APLCA: *Aplysia californica*: Q9NDE7 [10]; INS-3: *Caenorhabditis elegans*: Q09628 [9]: INS-17: *Caenorhabditis elegans*: G5EFH1 [9]. Cysteines highlighted in yellow and conserved residues bolded. Sequence alignments performed in Clustal Omega (https://www.ebi.ac.uk/Tools/msa/clustalo/, accessed on May 2020) [27,28,29].

The representative ILP full-length sequence IlO1_i1 (Figure 2a) from *Oulactis* sp. was selected for additional bioinformatic and functional studies. IlO1_i1 is composed of a signal peptide and B-, C-, and A-chains, with the full-length sequence having an *E*-value similarity of 1.7 × 10^−6^ to the insulin family. An NCBI-Blastp search of IlO1_i1 returned hits against sequences from six cnidarian genomes, including the sea anemones *Actinia tenebrosa* [42], *Exaiptasia pallida* [43] and *Nematostella vectensis* [44], along with three stony corals, *Acropora millepora* [45], *Stylophora pistallata* [46] and *Pocillopora damicornis* [47]. None of the six sequences returned in the NCBI blastp search has been functionally characterized (Supplementary Material, Table S1 and Figure S2). 

The aligned cnidarian sequences showed highly conserved residues in the A chain and more variability in the B chain. Four of the cnidarian sequences, including IlO1_i1, contained an additional cysteine pairing in the N terminus of the B chain (Supplementary Material Figure S2).

The predicted A- and B-chains of IlO1_i1 were aligned (Figure 2b,c) against human insulin and various characterized invertebrate ILPs, with the exception of the *Nematostella vectensis*, which is a predicted peptide. IlO1_i1 shares the same conserved cysteine arrangement reported previously in studies of ILPs [5]. The A- and B-chains share a highly conserved cysteine framework (CCx_[4]_Cx_[8]_C and Cx_[11]_C, respectively) with the exception of an additional two cysteines at the N-terminus of the B-chain in IlO1_i1. In addition, the Gly after Cys_1_ is wholly conserved in the B-chain.

The sequence similarity of human insulin and characterized invertebrate ILPs to IlO1_i1 was examined further using pairwise alignment (Table 1). As expected, IlO1_i1 has a much higher sequence similarity to cnidarian ILPs than to other invertebrate ILPs, with the lowest sequence similarity to *Caenorhabditis elegans* (INS-3 and INS-17). INS-3 and INS-17 are restricted in similarity only at the C-terminus of the A-chain, as opposed to INS_APLCA (Mollusc ILP), where the similarity occurs at the N-terminus of the A-chain (Table 1). Additionally, *Caenorhabditis elegans* sequences INS-3 and INS-17 do not possess B-chains and so were excluded from the subsequent analysis and alignment. Based on the percentage similarity of sequences alone, it is not possible to infer a function for IlO1_i1, as the most similar (characterized) ILPs are diverse in function, with roles in energy metabolism, inducement of a hypoglycemic coma, or growth development.

To ascertain the distribution and isoform variability of ILPs in *Oulactis* sp. and possible tissue-specific biological functions, the bioinformatic search for ILPs was extended to additional *Oulactis* sp. transcriptomes from discrete morphological regions, including mesenterial filaments and gametes, and the frill and acrorhagi. These morphological regions perform different biological functions from those of the tentacles, which are used primarily in defense and prey capture by the sea anemone [49,50]. It has been shown that venom expression varies between tentacles and other discrete regions in sea anemones [51].

Amino acid sequences meeting the bioinformatic search parameters were identified, and data were extracted. The mesenterial filament and gametic tissue returned only partial sequences so were excluded from further analysis. The frill and acrorhagi contained two unique ILPs. The tentacle transcriptomes from an additional two individuals contained seven unique sequences. These sequences did not match the amino acid sequence of IlO1_i1, but they all shared the highly conserved residues in the A-chain, while the B- and C-chains had minimal similarity. Additional sequences (ENA-EMBL Acc. No. OU729069-77) and alignments against IlO1_i1 may be found in the Supplementary Material (Figure S3). Two sequences were found in two individuals’ transcriptomes (tentacles, frill, and acrorhagi), indicating that there is some conservation of ILPs between tissues and individuals. Although no identical, translated ILP sequence was found in all five individuals, illustrating a high variability in the gene family among individuals.

### 3.2. Synthesis and Characterization

A truncated analogue of IlO1_i1 in which the additional cysteine pairing (Figure 2a) was omitted in order to mimic known active insulin and ILP sequences was synthesized by Fmoc solid-phase peptide synthesis [52] of the individual chains and subsequent regioselective disulfide bond formation [53,54]. Briefly, the two chains were separately chemically assembled using selective thiol protection on the Cys residues, and, after purification, sequential S-deprotection and simultaneous disulfide bond formation afforded the target peptide in an overall purified yield of 24%. Chemical characterization by RP-HPLC and MALDI-TOF MS confirmed the expected purity and composition of the peptide (Supplementary Materials, Figure S9).

### 3.3. Spectroscopic Studies

**Figure 3:**
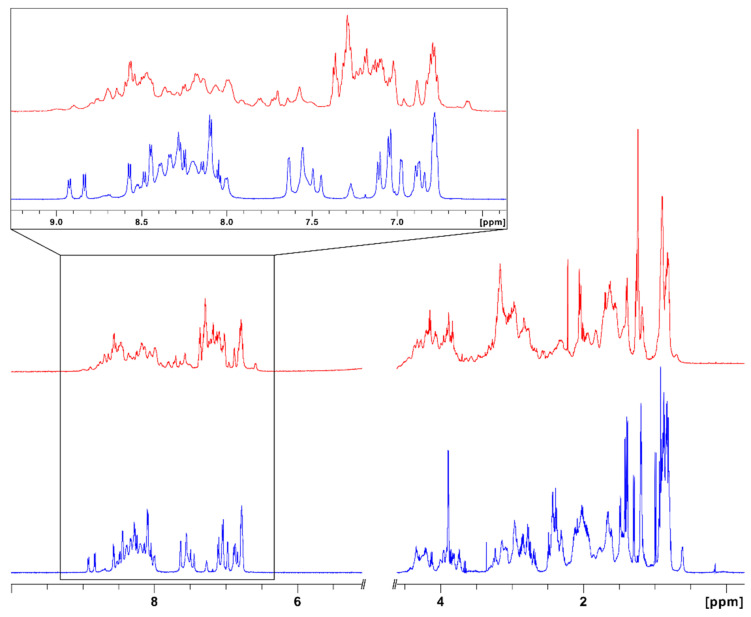
One-dimensional ^1^H NMR spectrum of the synthetic *Oulactis* sp. insulin-like peptide IlO1_i1 (blue). The spectrum was acquired at 298 K and pH 3.6 in 90% H_2_O/10% D_2_O on a Bruker Avance III 600 MHz spectrometer. The spectrum in red is from synthetic Con-Ins G1 [14], acquired at 298 K and pH 3.5. Expanded views of the amide-aromatic regions are shown in the inset.

The conformation of the synthetic peptide was assessed by ^1^H nuclear magnetic resonance (NMR) and circular dichroism (CD) spectroscopy. As shown in Figure 3, synthetic IlO1_i1 has a moderately well-dispersed ^1^H NMR spectrum, consistent with a folded structure [55,56]. Indeed, the spectral dispersion and resonance linewidths are comparable to those of Con-Ins G1, a representative vertebrate insulin from the cone snail *Conus geographus* [14], which is active on the human insulin receptor but monomeric in aqueous solution [57].

**Figure 4:**
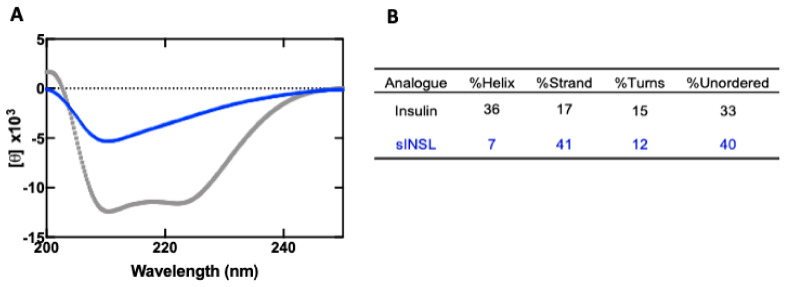
(**A**) CD spectra of insulin (grey) and IlO1_i1 peptide (blue). The percentages of helical content shown in the table (**B**) were calculated using the CDSSTR algorithm for deconvolution against the reference protein database set SMP180.

CD spectra (Figure 4), however, indicated that IlO1_i1 does not display the characteristic helical content of insulin (36% overall, Figure 4B) and is largely disordered, even though it has the canonical insulin disulfide connectivities.

### 3.4. Receptor Binding Assays

**Figure 5:**
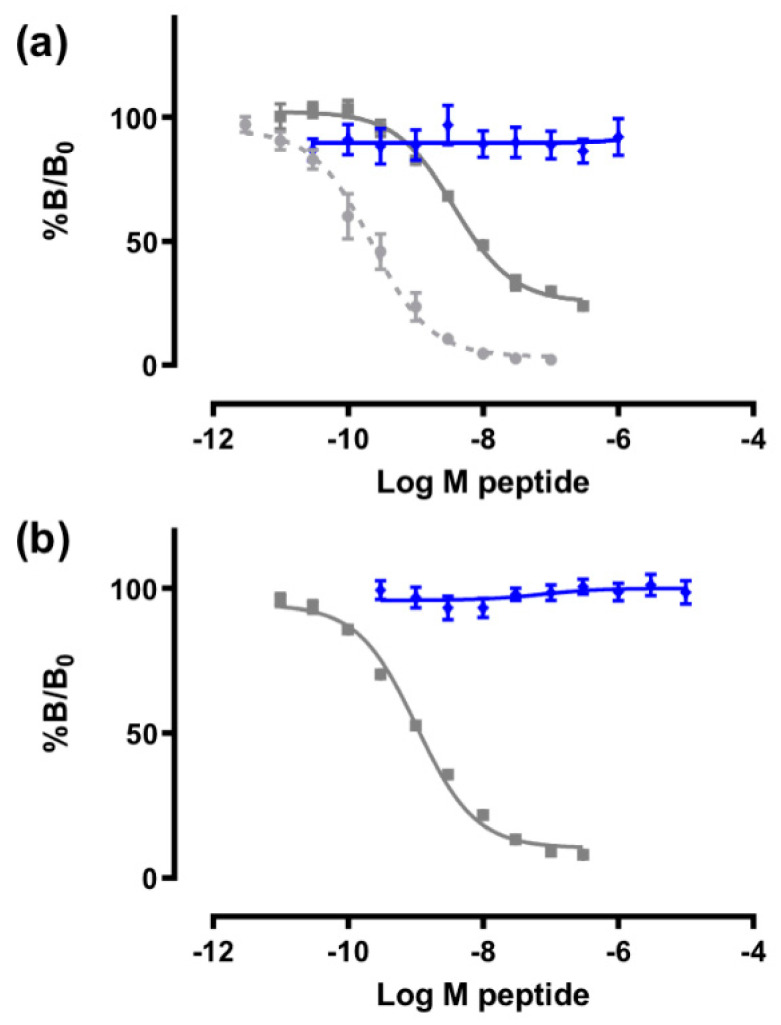
The ability of IlO1_i1 to bind to (**a**) IGF-1R and (**b**) IR-B was measured in competition binding assays using europium-labelled insulin for IR-B and europium-labelled IGF-1 for IGF-1R assays. Increasing concentrations of IlO1_i1 (blue), insulin (grey), and IGF-1 (grey dashed) were added. The results are expressed as a percentage of binding in the absence of competing ligand (%B/B_0_). Data shown are the mean ± S.E., with error bars shown where greater than the size of the symbols. n ≥ 3 independent experiments were conducted, each with triplicate technical replicates, except for IlO1_i1 binding IGF-1R, where n = 2.

Since previously characterized ILPs have been shown to bind to the insulin and/or IGF-1 receptors [16,58,59], the ability of IlO1_i1 to bind human IR (isoform B, IR-B) and IGF-1R was measured in competition binding assays using europium-labelled insulin and IGF-1, respectively (Figure 5). Insulin bound with high affinity to IR-B (IC_50_ = 1 nM), as reported previously [37]. However, even at 10^−5^ M concentration, IlO1_i1 was unable to compete with europium-labelled insulin bound to IR-B (Figure 5b). Similarly, both IGF-1 and, to a lesser extent, insulin were able to effectively compete for binding, as expected (IC_50_ 0.22 nM and 3.7 nM, respectively) [37]. However, IlO1_i1 did not compete with europium-labelled IGF-I binding to the IGF-1R (Figure 5a).

### 3.5. Potassium, Sodium, and Calcium Channel Assays

**Figure 6:**
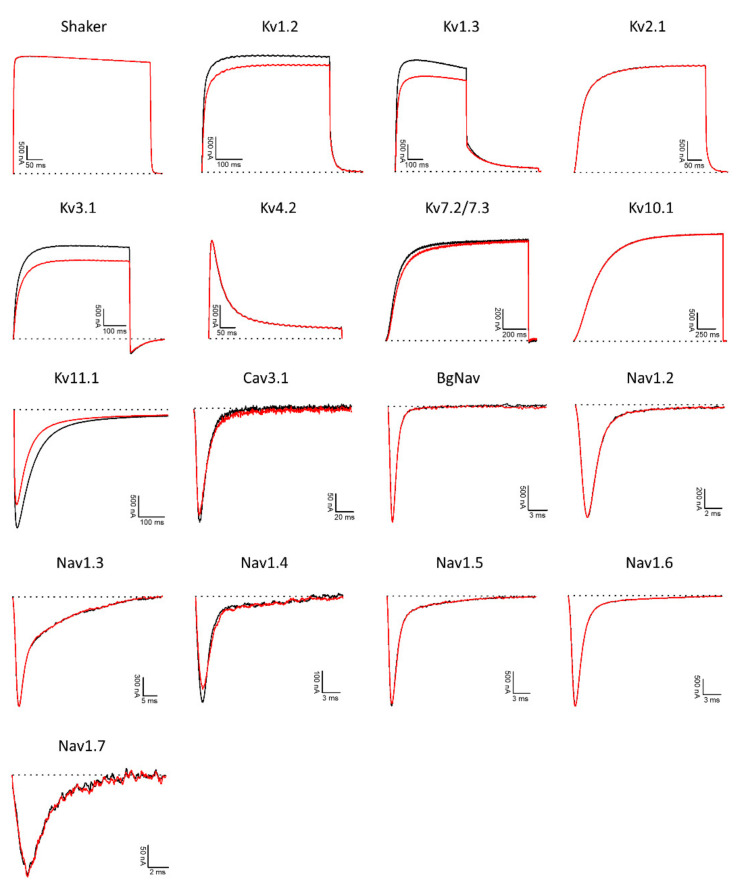
Electrophysiological screening of IlO1_i1 (8 µM) on potassium (K_V_), sodium (Na_V_), and calcium (Ca_V_) channels. The black lines represent the controls, while the red lines indicate the current obtained after the addition of the peptide. Dotted lines represent zero current level. The graphs illustrate the effects obtained in a series of at least three independent experiments (*n* ≥ 3).

As many disulfide-rich peptides from sea anemones and other marine organisms target ion channels [60,61] IlO1_i1 was tested on a panel of different voltage-gated ion channels, comprising potassium (K_V_), sodium (Na_V_), and calcium (Ca_V_) channels. At 8 µM, small inhibitory activity levels were observed on K_V_1.2 (3.6 ± 1.9%), K_V_1.3 (11.9 ± 0.9%), K_V_3.1 (12.7 ± 2.6%), K_V_11.1 (hERG) (30.5 ± 1.6%), and Na_V_ 1.4 (14.2 ± 2.2%) channels (Figure 6). Although only limited activity at a relatively high concentration was observed on these ion channels, insulin-like peptides may display different roles in fine-tuning specific physiologic functions governed by ion channel activity in certain target species. However, further studies would be needed to validate and better understand such hypotheses.

## 4. Discussion

The conserved cysteine spacing in the A- and B-chains of IlO1_i1 found in this study is consistent with ILPs sequences found in other invertebrates, with the C peptide being highly variable [5]. Based on the sequence similarity to insulin and previously characterized ILPs, we were unable to predict the function of IlO1_i1. It is becoming increasingly evident that sequence similarity alone is not a sufficient indicator of peptide functionality [62,63,64]. As more invertebrate ILPs are characterized with a range of functions relating to tissue-specific regions, it may become possible to more accurately predict which sequences are likely to have a particular functionality.

To date, no native ILP has been identified that falls outside the three basic structural types of α-type, β-type, or γ-type. Consequently, the finding that the full-length gene sequence for *Oulactis* sp. ILP (IlO1_i1) contains a B-chain N-terminal extension that includes a putative intra-chain disulfide bond raised the question of whether it is an anomaly (see below). We made the reasonable assumption that it was unlikely to constitute a native ILP structure and therefore undertook to chemically assemble and assess the biological activity of the canonical insulin sequence of this peptide.

The solid-phase synthesis of IlO1_i1 was straightforward using our well-established procedures that have been utilized previously to prepare numerous other insulin-like peptides and their analogues [53,54]. Each chain was readily prepared, the stepwise formation of each disulfide bond was efficient, and the overall yield of the resulting, highly purified IlO1_i1 was comparable to those of other insulin-like peptide syntheses.

In light of the fact that we elected to truncate the additional cysteine pairing to mimic known active insulin and ILPs, in future chemical synthesis studies it may be worthwhile to prepare full-length IlO1_i1. Synthesis of the four-disulfide analogue would allow a comparison of its functional profile with that of the canonical ILP analogue investigated here, as well as determination of whether the extended B-chain identified through bioinformatics forms a new class of ILP structure that occurs in Cnidaria.

Whilst it was expected that the CD spectra of IlO1_i1 would reveal a peptide with a high helical content, as is seen with most insulin-like peptides (including the insulins from cone snail venom and *Drosophila* insulin-like peptide 5 [16,58]), the high number of sequence differences from other known insulin-like peptides makes it difficult to predict helical content. The IlO1_i1 peptide lacks the key FFY motif at the end of the B-chain, which in human insulin engages with the high-affinity binding site on the IR. Cone snail insulin Con-Ins G1 also lacks this motif and yet binds with reasonable affinity. Structural and analogue studies of Con Ins G1 revealed that two tyrosine residues at positions B15 and B20 substitute for the lack of the FFY motif [16,57]. Despite the IlO1_i1 peptide having a tyrosine at B15 it is unable to bind to the IR, suggesting that other sequence differences prevent binding.

Owing to the weak activity found on the range of ion channels tested in this study, conducting additional assays on IlO1_i1, including metabolism and growth studies, would be of interest. It appears unlikely from the assays conducted here that this sea anemone ILP has a role in prey capture, which is in contrast to some cone snails that use insulin as part of their venom arsenal [14]. Characterized sequences that show the closest similarity to IlO1_i1 play a role in metabolism (human insulin) and growth development in the brain (bombyxin). However, sea anemones lack the traditional circulatory system by which vertebrates, or even other invertebrates, process energy; they also lack a central nervous system and brain [50,65]. Nonetheless, as animals they must still metabolize energy from food.

Some sea anemone species contain symbiotic zooxanthellae, enabling them to acquire energy via photosynthesis from the algae [65,66]. Food is linked to the growth and size of sea anemones, which can change dependent upon food availability, i.e., they can reduce in size during times of shortage [50,67,68]. There are relatively few studies on the metabolic function of sea anemones and the genes involved; additional studies on ILPs identified in this study may progress our knowledge in this area. Conversely, conducting assays on other characterized invertebrate sequences and determining their ion channel activity may clarify whether the weak activities of IlO1_i1 on K_V_1.2, 1.3, 3.1, and 11.1 channels, as well as Na_V_1.4, are an isolated occurrence or specific to sea anemones. It would also be informative to assess the activity of IlO1_i1 on channels from invertebrate species, especially those from the marine environment such as small shrimp and crabs, which form part of the sea anemone diet.

The synthesis of ILPs sourced from discrete tissue regions other than the tentacles may reveal that sea anemone ILPs have varied functional roles dependent upon the tissue of origin. For example, the synthesis and assay of ILPs located in the gametic material may reveal functions and roles aligned to sexual reproduction. We cannot, however, predict that such ILPs will serve a similar function in other Anthozoa. Reproduction strategies can be highly variable among genera, i.e., sexual (broadcast spawners) vs. asexual reproduction (e.g., fission or pedal disc laceration), with some species even utilizing a combination of both strategies [69], illustrating the complex nature of these animals.

In this study we determined that novel ILPs are present in sea anemone tissues that perform specific biological functions (e.g., tentacles for defense), and that different ILPs can be localized to specific tissues. Additionally, ILPs within the Anthozoa have a highly conserved A-chain and a B-chain with an additional pair of cysteines that may prove to be a new structural class of ILPs. Further research needs to be conducted to establish the function of ILPs in sea anemones and their sister taxa, and to determine whether their activities correlate with specific biological functions in their tissue of origin.

## Figures and Tables

**Table 1:** Pairwise similarity of the A- and B-chains of the sea anemone insulin-like protein (IlO1_i1) to human insulin and select characterized invertebrate ILPs, and tissue expression. Uniprot code supplied in parentheses under the ILP name in addition to the WormBase ID for *Caenorhabditis elegans*.

Insulin-like Protein (ILP)	Phylum/Class Species	Function/ Tissue Expression	A-Chain% Similarity	B-Chain% Similarity	Ref.
Nemve_207484 (A7S6C3)	Cnidaria/Anthozoa *Nematostella vectensis*	predicted ILP (larval whole animal)	61.5	40.9	[25,44]
Insulin (P010308)	Chordata/Mammalia *Homo sapiens*	metabolize energy (pancreas)	54.5	24.4	[33]
Con-Ins 1A (A0A0B5AC95)	Mollusca/Gastropoda *Conus geographus*	induce insulin coma (venom)	50.0	34.2	[14]
Bombyxin (Q17192)	Arthropoda/Insecta *Bombyx mori*	growth development (brain)	50.0	32.5	[8]
INS_APLCA (Q9NDE7)	Mollusca/Gastropoda *Aplysia californica*	metabolize energy (ganglia and cellular clusters)	31.4	28.8	[10]
INS-3 (Q09628; WBGene00002086)	Nematoda *Caenorhabditis elegans*	antagonist of the IIS ^1^ pathway (coelomocyte; egg-laying apparatus; gonad; head muscle; and nervous system)	23.4	-	[9,48]
INS-17 (G5EFH1; WBGene0000210)	Nematoda *Caenorhabditis elegans*	agonist of the IIS ^1^ pathway (egg-laying apparatus; gonad; head muscle; neurons; and somatic nervous system.)	23.1	-	[9,48]

^1^ insulin/insulin-like growth factor signaling.

## Supplementary Materials

The following are available online at https://www.mdpi.com/article/10.3390/biom11121785/s1, Figure S1: Alignment of full-length translated insulin-like protein (ILPs) sequences from the tentacle transcriptomes of three individuals of the sea anemone *Oulactis* sp., Figure S2: Sequence alignment of top six species hits in NCBI-Blastp of Insulin-like_Oulsp_1_i1 (IlO1_i1) from *Oulactis* sp., Figure S3: Sequence alignment of ILPs from five individuals of *Oulactis* sp. from two discrete morphological tissue regions: the tentacles and the frill and acrorhagi, Figure S4: Synthetic sea anemone insulin-like peptide [Cys7,12 (S-thiol), Cys8(But), Cys21(Acm)] A-chain, Figure S5: Synthetic sea anemone insulin-like peptide [Cys7,12 (S-S), Cys8(But), Cys21(Acm)] A-chain, Figure S6: Analytical RP-HPLC of sea anemone insulin-like peptide [Cys7,12 (S-S), Cys8(Pyr), Cys21(Acm)] A-chain, Figure S7: Synthetic sea anemone insulin-like peptide [Cys14 (S-thiol), Cys26(Acm)] B-chain, Figure S8: Synthetic sea anemone insulin-like peptide [Cys7,12 (S-S), Cys21(Acm)] A-chain/[Cys26(Acm)] B-chain, Figure S9: Sea anemone insulin-like peptide [Cys7,12 (S-S), Cys21(Acm)] A-chain/[Cys26(Acm)] B-chain, Table S1: Top 6 species in NCBI-Blastp hits for full-length insulin-like peptides (ILPs) against IlO1_i1.
