# Supplementary material for: Identification, Synthesis, Conformation and Activity of an Insulin-like Peptide from a Sea Anemone
Source: Biomolecules. 2021 Nov 29;11(12):1785. doi: 10.3390/biom11121785 (PMC8698791; doi:10.3390/biom11121785)
Supplement: Supplementary file 1 [file biomolecules-11-01785-s001.zip › biomolecules-1417293-supplementary.pdf]

Supplementary Information

Identification, synthesis, conformation and activity of an insulin-like peptide from a sea anemone

Michela L. Mitchell [1, 2, 3*], Mohammed Akhter Hossain [4, 5], Feng Lin [4], Ernesto L. Pinheiro-Junior [6], Steve Peigneur [6], Dorothy C. C. Wai [1], Carlie Delaine [7], Andrew J. Blyth [7], Briony E. Forbes [7], Jan Tytgat [6], John D. Wade [4, 5] and Raymond S. Norton [1,8 *]

1 Medicinal Chemistry, Monash Institute of Pharmaceutical Sciences, Monash University, 381 Royal Parade, Parkville, Victoria, 3052, Australia; michela.mitchell@qm.qld.gov.au; dorothy.wai@monash.edu; ray.norton@monash.edu
2 Sciences Department, Museum Victoria, G.P.O. Box 666, Melbourne, Victoria, 3001, Australia
3 Queensland Museum, PO Box 3000, South Brisbane 4101, Queensland, Australia;
4 Florey Institute of Neuroscience and Mental Health, University of Melbourne, Parkville, Victoria 3010, Australia; akhter.hossain@unimelb.edu.au; feng.lin@florey.edu.au; john.wade@florey.edu.au
5 School of Chemistry, University of Melbourne, Parkville, Victoria 3010, Australia;
6 Toxicology and Pharmacology, University of Leuven, O&N 2, Herestraat 49, PO Box 922, 3000, Leuven, Belgium; ernesto.lopes@kuleuven.be; steve.peigneur@kuleuven.be; jan.tytgat@kuleuven.be
7 Flinders Health and Medical Research Institute, Flinders University, Bedford Park, South Australia 5042, Australia.; carlie.delaine@flinders.edu.au; andrew.blyth@flinders.edu.au; briony.forbes@flinders.edu.au
8 ARC Centre for Fragment-Based Design, Monash University, Parkville, Victoria 3052, Australia
* Correspondence: ray.norton@monash.edu (RSN); michela.mitchell@qm.qld.gov.au (MLM)

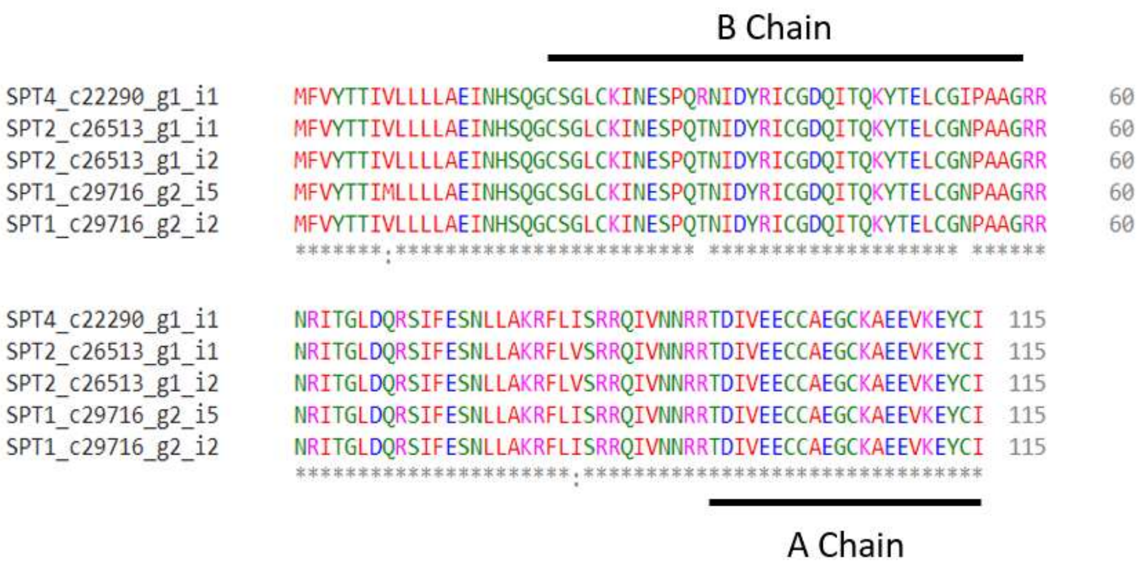

Figure S1. Alignment of full-length translated insulin-like peptide (ILP) sequences from the tentacle transcriptomes of three individuals of the sea anemone *Oulactis* sp. The gene is highly conserved across individuals of the species, particularly in the A chain. Sequences were deposited in the European Nucleotide Archive (EMBL-ENA) database under Project No. PRJEB34263 (Acc. No. LR700308–11). Sequence alignment was conducted in Clustal Omega (https://www.ebi.ac.uk/Tools/msa/clustalo/) [1–3]

```
Stylophora_pistillata_(PFX24051.1)          ---------------------------------------------------MMTP-----
Pocillopora_damicornis_(RMX49271.1)         ---------------------------------------------------MTTL-----
Acropora_millepora_(XP_029199002.1)         -------------------------------------------MYAAKSKMIKSCLWSG-
Nematostella_vectensis_(XP_032220951.1)     ----------------------------------------------MGCSKMDTT-----
Exaiptasia_pallida_(KXJ12931.1)             MFSSKRIVTAYGRPKNLKEILAPSKLRENMAISTSNSSIDRFLEISANYNMLDRPGMNL-
Actinia_tenebrosa_(XP_031575451.1)          -----------------------------MEVSNA----VSFTKFS--CSLLEKVRYSGE
Oulactis_sp_(IlO1_i1)                       ------------------------------------------------------------

Stylophora_pistillata_(PFX24051.1)          -----LSSRITILLYTCILTVGSNSKQSVPCAENCKVSEIPRRPISFRICGDQIMTAFKQ
Pocillopora_damicornis_(RMX49271.1)         -----LSNRITIILCMSILTAGSDSKQSIPCVENCKVNEIPRRPISFQVCGDQIVSAIKQ
Acropora_millepora_(XP_029199002.1)         -----VAVSAIFL--------LIGLTKGQEPQRLYKVNEVGNSLVDGKYCGDQIMQLFTH
Nematostella_vectensis_(XP_032220951.1)     ------IVSMVLLLIVSQLAVMVSGTCNAERTNCWKTSEAGHSQVDYHLCGDQISDAWTI
Exaiptasia_pallida_(KXJ12931.1)             RTMLFYFGLAVIFLI-----------DSSQTQTLYKVNEVGGSQVDRNLCGSDIPTAIKD
Actinia_tenebrosa_(XP_031575451.1)          NKMFIYTTIMLLLLAEI--------NHSQGCRGYCKINESPQTNIDYRICGDQIAQKYME
Oulactis_sp_(IlO1_i1)                       --MFVYTTIMLLLLAEI--------NHSQGCSGLCKINESPQTNIDYRICGDQITQKYTE
                                                       ::              .       * .*     :. . **.:*

Stylophora_pistillata_(PFX24051.1)          ICRIHARELRD-------GNEIFWEKRRASEFLT-----S--SGRFRRSYTVVEECCKEG
Pocillopora_damicornis_(RMX49271.1)         ICGIHAREMRD-------ARDLVWEKRRASKFLS-----S--TGRFRRSYTVVEECCIEG
Acropora_millepora_(XP_029199002.1)         YCFNQGRKRRSVF----------LNEQEATSFLLHNTQRHRRQASSSQATNVWEECCIEG
Nematostella_vectensis_(XP_032220951.1)     ICYGGGVTARQRQ-INRRDLSIVQSADEARQFN-----SKTQRGKRSSIYTITEECCVEG
Exaiptasia_pallida_(KXJ12931.1)             ICGIKKRQNIP-RKYGRDPNNILEKEEFAKRFL-----RVRR-----QTTDIVEECCVEN
Actinia_tenebrosa_(XP_031575451.1)          LCGNPRAGRRNHITHGLDHRSILESNSLAKRFL-----ISRRQP-NSQSTDIVEECCVEG
Oulactis_sp_(IlO1_i1)                       LCGNPAAGRRNRI-TGLDQRSIFESNLLAKRFL-----ISRRQIVNNRRTDIVEECCAEG
                                             *                      .   *  *                    : **** *.

Stylophora_pistillata_(PFX24051.1)          CVIEEMREYCY
Pocillopora_damicornis_(RMX49271.1)         CVIEELREYC-
Acropora_millepora_(XP_029199002.1)         CAHEEVKEYC-
Nematostella_vectensis_(XP_032220951.1)     CKQEEIREYC-
Exaiptasia_pallida_(KXJ12931.1)             CAIEEIAEYC-
Actinia_tenebrosa_(XP_031575451.1)          CRAEEVQEYC-
Oulactis_sp_(IlO1_i1)                       CKAEEVKEYCI
                                            *  **: ***
```

Figure S2. Sequence alignment of top six species hits from NCBI Blastp of insulin-like_Oulsp_1_i1 (IlO1_i1) from *Oulactis* sp. E-value threshold 1e-10. Alignment shows high sequence similarity and conservation of the A chain, very low similarity in the B Chain with many of the sequences lacking obvious cleavage sites for the B and C chains. Sequence alignment was performed in Clustal Omega (https://www.ebi.ac.uk/Tools/msa/clustalo/) [1–3].

```
Tentacle_ILP_Oulsp_3_i1         MGSFSRCFLPILIFLLFLVCLTL----ARP--KGRGRKMYKIRETGGKAVSFRVCGDYTY   54
Tentacle_ILP_Oulsp_8_i1         --MFSEKSLHVL-RVFLVLCL-LLHLVSAT--CNSKGQCWKTSEAPNKSVDFHLCGSMIP   54
Frill_Acror_ILP_Oulsp_9_i1      --MFSEKSLNVL-RVLLVLCL-LLHLVSAT--CNSKGQCWKTSEAPNKSVDFHLCGSMIP   54
Tentacle_ILP_Oulsp_4_i1         --MFSEKSLNVL-RVLLVLCL-LLHLVSAT--CNSKGQCWKTSEAPNKSVDFHLCGSMIP   54
Frill_Acror_ILP_Oulsp_10_i1     ----MDICVKTL-RLAFVLTLAFSVALSQN--CLMRGGCHKVEESSRSQVDFFLCGDSIY   53
Tentacle_ILP_Oulsp_6_i1         ----MDICVKTL-RLAFVLTLAFSVALSQN--CLMRGGCHKVEESSRSQVDFFLCGDSIY   53
Tentacle_ILP_Oulsp_7_i1         ----MDICVKTL-QLAFLLTLAFSVALSQN--CLMRGGCHKVEESSRSQVDFFLCGDSIY   53
Tentacle_ILP_Oulsp_5_i1         --------MASRYHTIHALSILLLVLIYQA---------YHVTSTPKSGKIDKYCGEQFI   43
Tentacle_ILP_Oulsp_5_i2         --------MASRYHTIHALSILLLVLIYQA---------YHVTSTPKSGKIDKYCGEQFI   43
Tent_Il01_i1                    ---------------MFVYTTIMLLLLAEINHSQGCSGLCKINESPQTNIDYRICGDQIT   45
                                                      :                 :  .:  .      **.

Tentacle_ILP_Oulsp_3_i1         ILFTSICMPRNYK--------RSSIDFLTDERTANDYLSGR----TGVTRRNYNIVEECC   102
Tentacle_ILP_Oulsp_8_i1         FAVRDICNIMMAAYGRRKQKARKSSGTIVYKNPNN---AKR----FRIAKRKFNIVEECC   107
Frill_Acror_ILP_Oulsp_9_i1      FAVRDICNIMMAAYGRRRHKARKSSGTIVYKNPNK---AKR----FRIAKRKFNIVEECC   107
Tentacle_ILP_Oulsp_4_i1         FAVRDICNIMMAAYGRRRHKARKSSGTIVYKNPNN---AKR----FRIAKRKFNIVEECC   107
Frill_Acror_ILP_Oulsp_10_i1     YTFMDVCKIKKRR----KREVKAAVNILVNRKEST---KFL----SRRKRRTYDIVEECC   102
Tentacle_ILP_Oulsp_6_i1         YTFMDVCKIKKRR----KREVKAAVNILVNRKEST---KFL----SRRKRRTYDIVEECC   102
Tentacle_ILP_Oulsp_7_i1         YTFIDVCKIKKRR----KREVKAAVNILVNRRESS---KFL----SRRKRRTYDIVEECC   102
Tentacle_ILP_Oulsp_5_i1         SAWSVCCGNNPKC-ENSKRQVQDEDDILTLPMFAKRFLLSRRQLSSINKRGSTDAVEECC   102
Tentacle_ILP_Oulsp_5_i2         SAWSVCCGNNPKC-ENSKRQVQDEDDILTLPMFAKRFLLSRRQLSSINKRGSTDAVEECC   102
Tent_Il01_i1                    QKYTELCGNPAAG-RRNRITGLDQRSIFESNLLAKRFLISRRQIVN---NRRTDIVEECC   101
                                      *                  . :      .              .   : *****

Tentacle_ILP_Oulsp_3_i1         LEGCVVEEVKEYCK---      116
Tentacle_ILP_Oulsp_8_i1         TEGCLVEEVREYC----      120
Frill_Acror_ILP_Oulsp_9_i1      TEGCIVEEVREYC----      120
Tentacle_ILP_Oulsp_4_i1         TEGCIVEEVREYC----      120
Frill_Acror_ILP_Oulsp_10_i1     HEGCRVEEVHEYCYVML      119
Tentacle_ILP_Oulsp_6_i1         HEGCRVEEVHEYCYVML      119
Tentacle_ILP_Oulsp_7_i1         HEGCRVEEVHEYCHVML      119
Tentacle_ILP_Oulsp_5_i1         NEGCSLSEIAEYQC---      116
Tentacle_ILP_Oulsp_5_i2         NEGCSLSEIAEYQC---      116
Tent_Il01_i1                    AEGCKAEEVKEYCI---      115
                                 ***  .*:  **
```

Figure S3. Sequence alignment of ILPs from five individuals of *Oulactis* sp. from two discrete morphological tissue regions: tentacles; frill and acrorhagi. The mesenterial filaments and gametic material bioinformatic analysis returned fragmented ILP sequences, which were excluded for subsequent analysis. Sequence alignment was conducted in Clustal Omega (https://www.ebi.ac.uk/Tools/msa/clustalo/) [1–3]. Sequences were deposited in European Bioinformatics Institute - European Nucleotide Archive (EMBL-ENA) database under Project No. PRJEB34263 (Acc. No. OU729069-77).

A

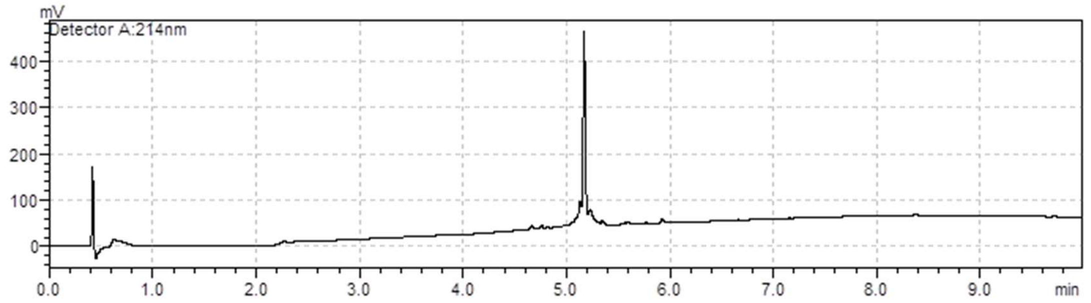

B

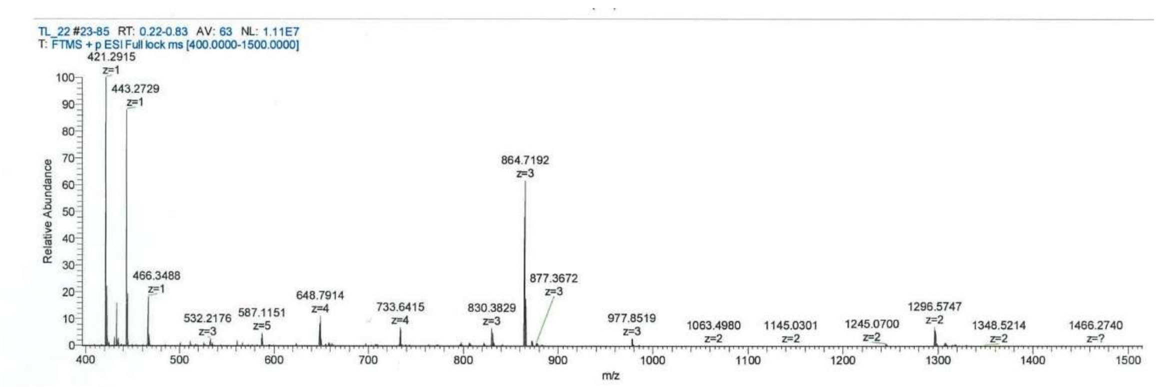

Figure S4. Synthetic sea anemone insulin-like peptide [Cys7,12 (S-thiol), Cys8(But), Cys21(Acm)] A-chain. (A) Analytical RP-HPLC. Column: Shimadzu Shim-Pack C18 (3.0 x 75 mm). Buffer A: 0.1% aqueous TFA; Buffer B: 0.1% TFA in CH3CN. Gradient: 0-90%B in 10 mins. Wavelength: 214 nm. Flow rate: 1.2 ml/min. Column temperature: 55°C. (B) ESI mass spectrometry. Calcd. [MH+] 2591.77, found [M/2+H]=1296.57, [M/3+H]=864.72).

A

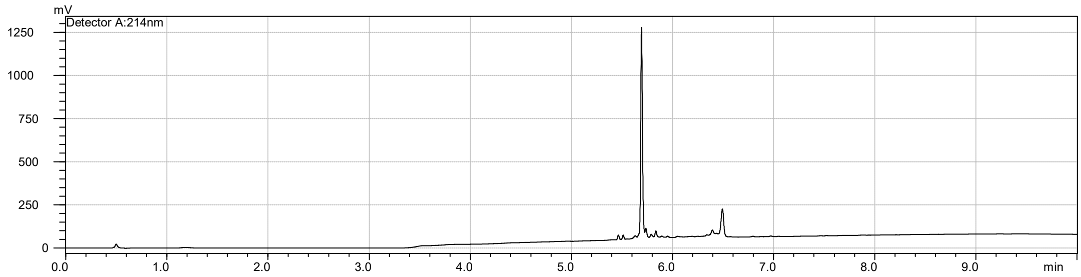

Figure S5. Synthetic sea anemone insulin-like peptide [Cys7,12 (S-S), Cys8(But), Cys21(Acm)]-A-chain. (A) Analytical RP-HPLC. Column: Shimadzu Shim-Pack C18 (3.0 x 75 mm). Buffer A: 0.1% aqueous TFA; Buffer B: 0.1% TFA in CH3CN. Gradient: 0-90%B in 10 mins. Wavelength: 214 nm. Flow rate: 1.2 ml/min. Column temperature: 55°C.

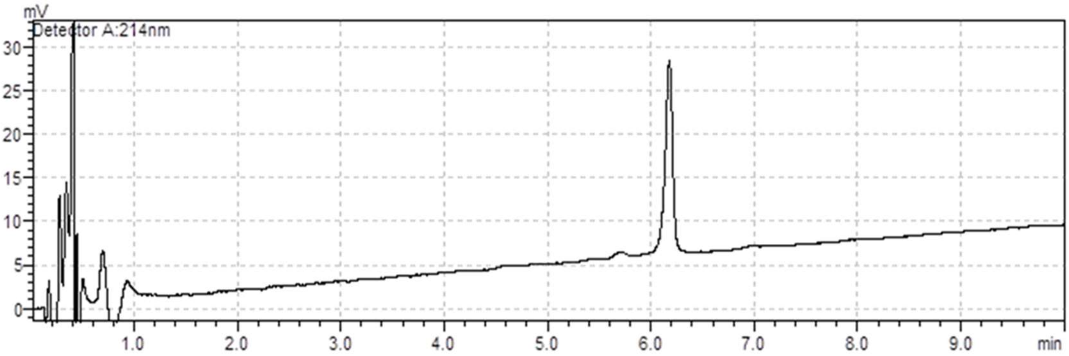

Figure S6. Analytical RP-HPLC of sea anemone insulin-like peptide [Cys7,12 (S-S), Cys8(Pyr), Cys21(Acm)] A-chain. Column: Shimadzu Shim-Pack C18 (3.0 x 75 mm). Buffer A: 0.1% aqueous TFA; Buffer B: 0.1% TFA in CH3CN. Gradient: 0-90%B in 10 mins. Wavelength: 214 nm. Flow rate: 1.2 ml/min. Column temperature: 55˚C.

A

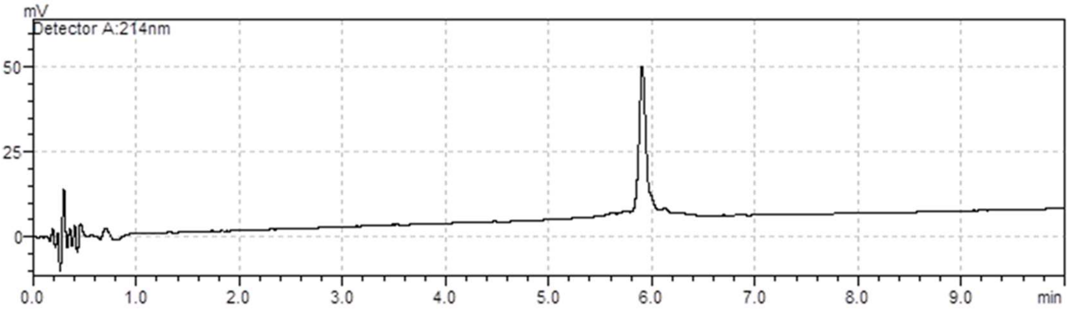

B

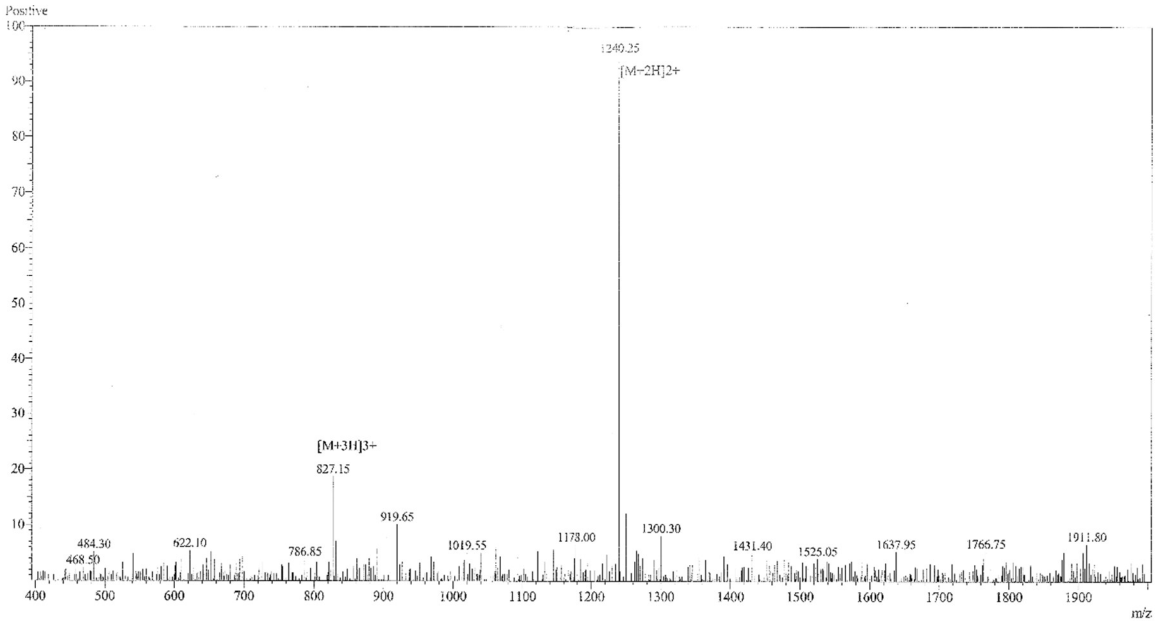

Figure S7. Synthetic sea anemone insulin-like peptide [Cys14 (S-thiol), Cys26(Acm)] B-chain. (A) Analytical RP-HPLC. Column: Shimadzu Shim-Pack C18 (3.0 x 75 mm). Buffer A: 0.1% aqueous TFA; Buffer B: 0.1% TFA in CH_3CN. Gradient: 0-90%B in 10 mins. Wavelength: 214 nm. Flow rate: 1.2 ml/min. Column temperature: 55˚C. (B) ESI mass spectrometry. Calcd. [MH+] 2480.50, found [M/2+H]=1240.35, [M/3+H]=827.15).

A

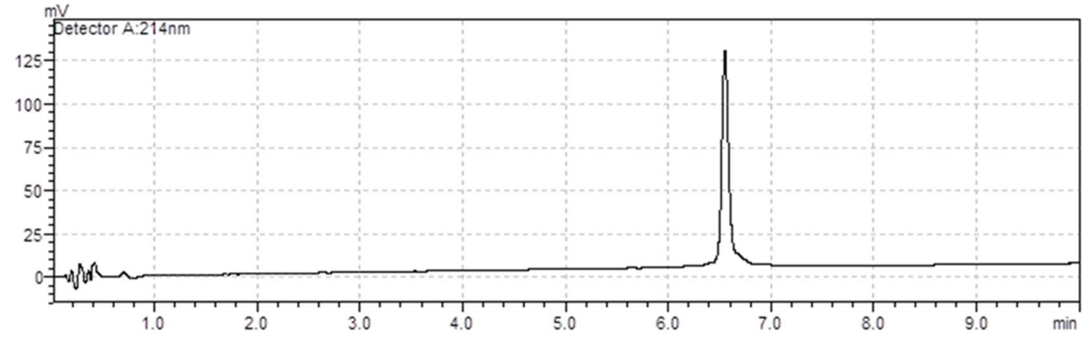

B

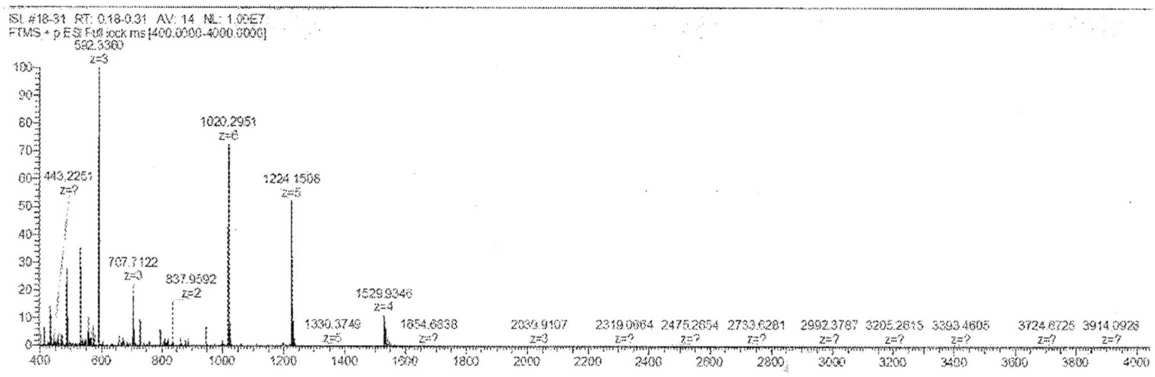

Figure S8. Synthetic sea anemone insulin-like peptide [Cys7,12 (S-S), Cys21(Acm)]A-chain/[Cys26(Acm)] B-chain. (A) Analytical RP-HPLC. Column: Shimadzu Shim-Pack C18 (3.0 x 75 mm). Buffer A: 0.1% aqueous TFA; Buffer B: 0.1% TFA in CH_3CN. Gradient: 0-90%B in 10 mins. Wavelength: 214 nm. Flow rate: 1.2 ml/min. Column temperature: 55°C. (B) ESI mass spectrometry. Calcd. [MH+] 6114.62, found [M/4+H]= 1529.93, [M/5+H]=1224.15, [M/6+H]=1020.30, [M/7+H]=1224.15).

A

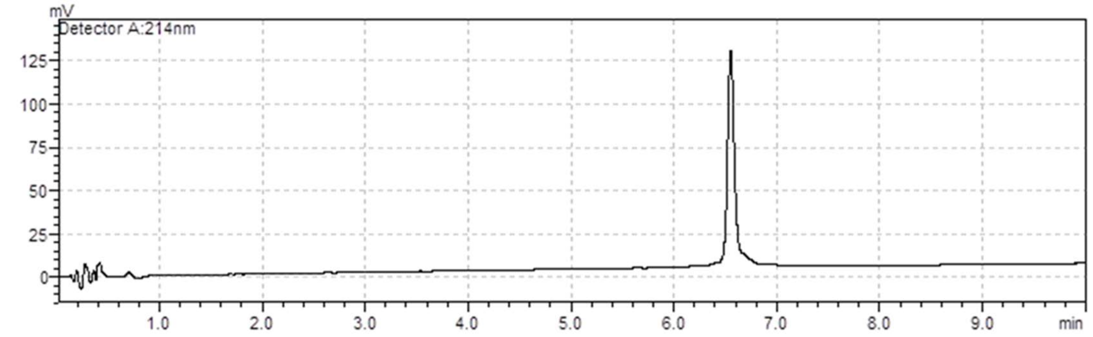

B

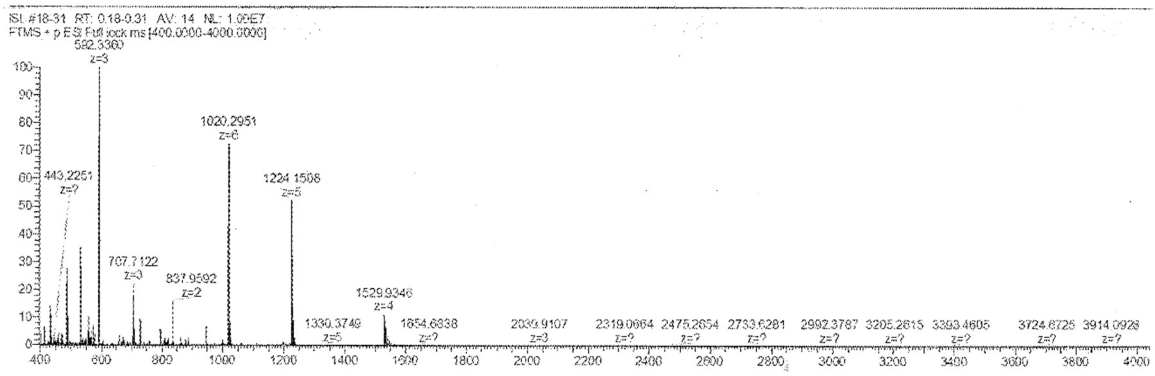

Figure S9. Sea anemone insulin-like peptide [Cys7,12 (S-S), Cys21(Acm)]A-chain/[Cys26(Acm)] B-chain. (A) Analytical RP-HPLC. Column: Shimadzu Shim-Pack C18 (3.0 x 75 mm). Buffer A: 0.1% aqueous TFA; Buffer B: 0.1% TFA in CH3CN. Gradient: 0-90%B in 10 mins. Wavelength: 214 nm. Flow rate: 1.2 ml/min. Column temperature: 55˚C. (B) ESI mass spectrometry. Calcd. [MH+] 6114.62, found [M/4+H]=1529.93 [M/5+H]=1224.15, [M/6+H]=1020.30, [M/7+H]=1224.15.

Table S1. Top six species hits (NCBI-Blastp) of full-length insulin-like proteins (ILPs) against IlO1_i1. The translated ILP Insulin-like_Oulsp_1_i1 (IlO1_i1) from the sea-anemone *Oulactis* sp. was searched against the NCBI-non-redundant database using the online Blastp search function.

**Species**	**% Identity**	**E-value**
Actinia tenebrosa (sea-anemone)	83	2e-61
Acropora millepora (stony coral)	38	9e-13
Exaiptasia pallida (sea-anemone)	37	2e-12
Stylophora pistillata (stony coral)	37	6e-11
Nematostella vectensis (sea-anemone)	35	2e-10
Pocillopora damicornis (stony coral)	37	3e-10

References

1. Sievers, F.; Wilm, A.; Dineen, D.; Gibson, T.J.; Karplus, K.; Li, W.; Lopez, R.; McWilliam, H.; Remmert, M.; Söding, J.; et al. Fast, scalable generation of high-quality protein multiple sequence alignments using Clustal Omega. *Mol. Syst. Biol.* **2011**, *7*, 539, doi:10.1038/msb.2011.75.

2. Goujon, M.; McWilliam, H.; Li, W.; Valentin, F.; Squizzato, S.; Paern, J.; Lopez, R. A new bioinformatics analysis tools framework at EMBL-EBI. *Nucleic Acids Res.* **2010**, *38*, W695–W699, doi:10.1093/nar/gkq313.

3. McWilliam, H.; Li, W.; Uludag, M.; Squizzato, S.; Park, Y.M.; Buso, N.; Cowley, A.P.; Lopez, R. Analysis tool web services from the EMBL-EBI. *Nucleic Acids Res.* **2013**, *41*, W597–W600, doi:10.1093/nar/gkt376.
